# Tecido Adiposo Epicárdico nos Fenótipos de Insuficiência Cardíaca – Uma Metanálise

**DOI:** 10.36660/abc.20200755

**Published:** 2022-01-11

**Authors:** Eduardo Thadeu de Oliveira Correia, Letícia Mara dos Santos Barbetta, Orlando Santos da Costa, Pedro el Hadj de Miranda, Evandro Tinoco Mesquita

**Affiliations:** 1 Universidade Federal Fluminense Niterói RJ Brasil Universidade Federal Fluminense , Niterói , RJ - Brasil

**Keywords:** Insuficiência Cardíaca/fisiopatologia, Pericardio/diagnóstico por imagem, Tecido Adiposo, Citotoxinas, Metanálise

## Abstract

**Fundamento:**

O tecido adiposo epicárdico (TAE) é aumentado em comorbidades comuns na insuficiência cardíaca (IC). Dessa forma, o TAE teria o potencial de mediar efeitos que levam à deterioração da função cardíaca.

**Objetivos:**

Esta metanálise tem o objetivo de investigar se a quantidade de TAE em todos os tipos de IC e cada tipo de IC são significativamente diferentes dos pacientes de controle.

**Métodos:**

Esta metanálise seguiu as diretrizes da *Meta-analysis of Observational Studies in Epidemiology* (Metanálise de estudos observacionais em epidemiologia). A pesquisa foi realizada nos bancos de dados MEDLINE, Embase e Lilacs até novembro de 2020. Dois autores realizaram a triagem, a extração de dados e a avaliação de qualidade. Um p-valor <0,05 foi definido como estatisticamente significativo.

**Resultados:**

Foram incluídos oito estudos observacionais, compreendendo 1248 pacientes no total, dos quais 574 eram de controle, 415 tinham IC com fração de ejeção reduzida (ICFER) e 259 tinham IC com fração de ejeção de faixa média ou preservada (ICFEfm ou ICFEP). A quantidade de TAE não era diferente entre todos os tipos de IC e o grupo de controle (DMP = -0,66, IC 95%: -1,54 a 0,23, p =0,14) . Analisando cada fenótipo de IC separadamente, pacientes com ICFER tinham TAE reduzido em comparação aos pacientes de controle (DMP = 1,27, IC 95%: - 1,87 a -0,67, p <0,0001), enquanto os pacientes com ICFEfm ou ICFEP tiveram TAE aumentado em comparação aos pacientes de controle (DMP = 1,24, IC 95%: 0,99 a 1,50, p <0,0001).

**Conclusão:**

A quantidade de TAE não era significativamente diferente entre todos os tipos de IC e o grupo de controle. Em pacientes com ICFER o volume de TAE era reduzido, enquanto em pacientes com ICFEP e ICFEfm, a quantidade de TAE era significativamente aumentada. Número de registro PROSPERO: CRD42019134441.

## Introdução

O tecido adiposo epicárdico (TAE) é um depósito de gordura viceral localizada ao redor do miocárdio.^1 , 2^ O TAE secreta várias quimiocinas e citocinas pró-inflamatórias, coletivamente chamadas de adipocinas.^3^ Além disso, devido à relação próxima entre TAE e miocárdio, a gordura epicárdica pode promover efeitos inflamatórios locais e mecânicos no músculo cardíaco e vasos coronários.^4^

Também se sabe que o TAE é aumentado em doenças sistêmicas que podem promover um estado pró-inflamatório sistêmico, tais como a obesidade e o diabetes, comuns em pacientes com insuficiência cardíaca (IC), especialmente em IC com fração de ejeção preservada (ICFEP).^5 - 7^ Dessa forma, o TAE pode mediar efeitos deletérios no miocárdio que pode levar a uma função cardíaca deteriorada.^8^

Estudos anteriores demonstram que o TAE é mais baixo em pacientes com IC do que em pacientes saudáveis.^9^ Entretanto, um estudo recente publicado por van Worden et al., que submeteu 64 pacientes com IC com fração de ejeção de faixa média (ICFEfm) ou ICFEP a ressonância magnética cardíaca (RMC), tinham volume de TAE mais alto do que os pacientes do grupo de controle.^4^

Apesar da relevância dessa associação, não há análises sistemáticas ou metanálises que ponderam as evidências disponíveis e trazem conclusões e discussões sobre este tópico. Dessa forma, o presente estudo tem o objetivo de realizar uma metanálise para investigar a associação entre TAE e cada fenótipo de IC.

## Métodos

Foi realizada uma metanálise utilizando-se os critérios definidos pelas recomendações do grupo *Meta-analysis of Observational Studies in Epidemiology* (MOOSE - Metanálise de estudos observacionais em epidemiologia).^10^ O protocolo desta metanálise foi registrado no PROSPERO sob o número CRD42019134441.

### Estratégias de pesquisa

Dois investigadores (ETOC, LMSB) pesquisaram, nos bancos de dados MEDLINE, Lilacs, e Embase, estudos que investigavam TAE em pacientes com IC, até novembro de 2020. A estratégia de pesquisa foi formada por uma combinação de termos em inglês e descritores do Medical Subject Headings (MeSH), consistindo em quatro palavras-chave *[(epicardial adipose OR epicardial fat) AND (heart failure OR cardiac insufficiency)]* , ou seja, [(adiposo epicárdico OU gordura epicárdica) E (falência cardíaca OU insuficiência cardíaca)]. Também foi utilizada uma pesquisa manual de referências para identificar possíveis estudos para inclusão. Cada título e resumo eram analisados independentemente pelos dois investigadores, que selecionaram os artigos que seriam relevantes para a análise. Depois disso, os textos completos dos artigos restantes foram analisados para selecionar os que seriam incluídos na análise quantitativa. Em caso de discordância, a decisão era tomada por meio de discussão e consenso entre os autores.

### Critérios de inclusão

Para se qualificarem, os estudos precisavam atender aos seguintes critérios: 1) População: o estudo incluía sujeitos humanos portadores de IC; 2) Intervenção: o estudo media o TAE e a fração de ejeção ventricular esquerda (FEVE) por tomografia computadorizada, ecocardiograma ou RMC; 3) Grupo de comparação: o estudo incluía pacientes sem IC; 4) Resultados: o estudo relatava médias e intervalos de confiança (IC) de 95% ou desvio padrão de TAE em pacientes com IC e o grupo de controle. 5) Desenho do estudo: o estudo era observacional.

### Extração de dados

A extração de dados foi realizada por dois investigadores (PMH, OSC), utilizando um formulário padronizado, e um terceiro autor (ETOC) realizou a verificação cruzada. Os dados extraídos incluíram: 1) Sobrenome do primeiro autor, ano de publicação; 2) Características dos estudos incluídos: número de pacientes, país onde o estudo foi realizado, braços do estudo, etiologia da IC, classe da New York Heart Association dos pacientes incluídos, fração de ejeção dos pacientes incluídos, índice de massa corporal, método de medição do TAE e principais achados 3) Resultados: médias, IC 95% e desvio padrão da TAE em pacientes com IC e grupo de controle.

### Avaliação da qualidade

O risco de viés nos estudos foi avaliado pela *Newcastle - Ottawa Quality Assessment Scale Case Control Studies* (Escala Newcastle-Ottawa para avaliação de qualidade de estudos caso controle), que analisa a seleção dos participantes em cada estudo, a comparabilidade dos casos e controles, e a exposição. A avaliação da qualidade foi realizada por dois investigadores (PMH, OSC), utilizando um questionário padronizado, e, em caso de divergência, chegava-se a uma conclusão por consenso. A qualidade dos estudos foi considerada boa se a pontuação fosse 7 ou 8, satisfatória se a pontuação fosse 5 ou 6, e insatisfatória se a pontuação ficasse entre 0 e 4 pontos. A avaliação da qualidade dos estudos incluídos está apresentada nas Tabelas 1 e 2.

**Tabela 1:** – Características dos estudos incluídos

Estudo	País	Definição de nº de IC e/ou subtipos de nº de IC	Definição do grupo de controle	Método de medição	Qualidade
Doesch et al.^12^	Alemanha	Presença de FEVE ≤35% no ecocardiograma e sinais e sintomas de IC. Esses pacientes foram classificados como IC devido a CMI ou CMD.	Pacientes saudáveis	RMC	Boa
Doesch et al.^9^	Alemanha	Pacientes com FEVE >50% no ecocardiograma na ausência de DAC significativa (estenose da artéria coronária ≥ 50% ou histórico de revascularização coronária ou IM prévio).	Pacientes saudáveis	RMC	Boa
Doesch et al.^7^	Alemanha	Histórico de IC sintomático e FEVE ≤35% no ecocardiograma. A etiologia isquêmica foi definida como a presença de algum vaso coronário epicárdico com ≥75% de estenose ou histórico de IM ou revascularização coronária. A CMD foi baseada no diâmetro diastólico final >56 mm e uma angiografia coronária normal realizada nos últimos 6 meses.	Pacientes saudáveis	RMC	Boa
Flüchter et al.^13^	Alemanha	Pacientes com IC prévia subclassificada em etiologias isquêmicas e dilatadas.	Pacientes saudáveis	RMC	Boa
Obokata et al.^14^	Estados Unidos	A ICFEP foi definida por sintomas clínicos de IC, FEVE >=50%, elevação das pressões de enchimento do VE medida diretamente (em repouso>15mmHg e/ou com exercício ≥25mmHg). ICFEP de não obesos por IMC <30kg/m^2^. ICFEP de obesos foi definida pela presença de obesidade grau II ou acima (IMC ≥35kg/m^2^).	Pacientes não obesos sem IC	Ecocardiograma	Boa
Khawaja et al.^15^	Estados Unidos	Pacientes com FEVE ≤55%. Este grupo foi subdividido em pacientes com disfunção moderada do VE (FEVE de 35% a 55%) e um grupo com disfunção grave do VE (FEVE ≤55%).	Pacientes sem histórico de IC ou disfunção do VE no ecocardiograma	TC	Boa
Tabakci et al.^16^	Turquia	CDNI foi definida como FEVE ≤45 com artérias epicárdicas coronárias normais vistas na angiografia.		RMC	Boa
van Woerden et al.^4^	Holanda	Pacientes com FEVE >= 40% no ecocardiograma, NT-proBNP >125ng/L e evidências ecocardiográficas de hipertrofia do VE, disfunção diastólica do VE, ou dilatação do átrio esquerdo de acordo com critérios de ESC. ICFEfm foi definida por FEVE entre 40 e 50, e ICFEP foi definida por FEVE >50%.	Pacientes saudáveis	RMC	Boa

*IMC; índice de massa corporal; DAC: doença arterial coronariana; RMC: ressonância magnética cardíaca; TC : tomografia computadorizada; CMD: cardiomiopatia Dilatada; IC: insuficiência Cardíaca; ICFEfm: insuficiência cardíaca com fração de ejeção na faixa média; ICFEP: insuficiência cardíaca com fração de ejeção preservada; ICFER: insuficiência cardíaca com fração de ejeção reduzida; CMI: cardiomiopatia isquêmica; VE: Ventrículo esquerdo; FEVE: fração de ejeção ventricular esquerda; IM: infarto do miocárdio; CDNI: cardiomiopatia dilatada não isquêmica; NT-proBNP: pró-peptídeo natriurético cerebral n-terminal; NYHA: New York Heart. Association.*

**Tabela 2:** – Características dos estudos incluídos

Estudo	Braços	N	Idade, anos	Sexo masculino (%)	IMC	FE	TAE| volume, peso ou espessura
Doesch et al.^12^	IC (FE ≤35%) Controles	41 16	63 ± 12 61 ± 11	88 75	27 ± 4 28 ± 5	27 ± 9 57 ± 6	44 ± 11 (g) 67 ± 10 (g)
Doesch et al.^9^	CMD (FE 35-50%) CMD (FE ≤35%) Todo CMD Controles	28 84 112 48	57,2 ± 13,4 60,1 ± 14,0 59,4 ± 13,9 60,9 ± 9,8	79 77 78 77	26,6 ± 4,6 27,3 ± 4,8 27,2 ± 4,7 27,3 ± 6,0	43,6 ± 6,9 23,0 ± 6,7 58,7 ± 5,2 58,7 ± 5,2	50,0 ± 21,9 (ml) / 25,0 ± 10,4 (ml/m^2^) / 47,0 ± 20,6 (g) / 23,5 ± 9,8(g/m^2^) 50,2 ± 13,9 (ml) / 25,7 ± 7,0 (ml/m^2^) / 47,2 ± 13,1 (g) / 24,2 ± 6,6 (g/m^2^) 50,2 ± 16,2 (ml) / 25,5 ± 8,0 (ml/m^2^) / 47,2 ± 15,2 (g) / 24,0 ± 7,5 (g/m^2^) 66,0 ± 15,3 (ml) / 33,5 ± 6,4 (ml/m^2^) / 62,1 ± 14,4 (g) / 31,7 ± 5,6 (g/m^2^)
Doesch et al.^7^	IC| Controles	66 32	63 ± 12 57 ± 11	82 78	27 ± 4 28 ± 4	27 ± 9 58 ± 5	46 ± 11 (ml) / 43 ± 11 (g) / 24 ± 5 (ml/m^2^) 71 ± 13 (ml) / 67 ± 13 (g) / 36 ± 5 (ml/m^2^)
Flüchter et al.^13^	IC Controles	43 28	61,9±12,4 56,6 ± 10,9	81,3 78,6	27,0 ± 4,4 27,5 ± 4,2	25,9 ± 6,8 58,8 ± 4,2	51,0 ± 20,9 g / 3,5 ± 1,5 (mm)- (Eixo longo) / 2,9 ± 1,3 (mm)-(Eixo curto) /3,2 ± 1,2(mm)- (Eixo longo/curto) 64,6 ± 21,2 g / 3,8 ± 1,5 (mm)- (Eixo longo) / 4,3 ± 1,3 (mm)-(Eixo curto) /4,1 ± 1,1(mm)- (Eixo longo/curto)
Obokata et al.^14^	Controles Não obesos ICFEP (FE>50%) Obesos ICFEP (FE>50%)	71 96 99	62 ± 10 70 ± 10 65 ± 11	42 36 36	25,4 ± 2,8 26,0 ± 2,7 40,8 ± 5,6	63 ± 4 63 ± 6 63 ± 6	6 ± 2 mm / 632 (517 - 768) ml 7±2 mm / 797 (643 - 979) ml 10±2 mm / 945 (831 - 1105) ml
Khawaja et al.^15^	IC (FE<55%) IC (FE 35-55%) IC (FE≤35%) Controles	60 43 17 321	54,2 ± 12,2 53,4 ± 12,2 59,8 ± 14,4 55,8 ± 10,4	19 35 53 70	30,7 ± 11,5 29,5 ± 4,7 31,3 ± 13,6 29,6 ± 6,7	NR NR NR NR	83,5 ± 67,1 (cm^3^) 96,1 ± 73,9 (cm^3^) 52,2 ± 29,7 (cm^3^) 114,5 ± 98,5 (cm^3^)
Tabakci et al.^16^	CMD Controles	93 38	49,9 ± 13,9 51,1 ± 10,0	69 61	27,7 ± 3,3 28,3 ± 3,4	32,0 ± 8,5 62,9 ± 4,9	4,1 ± 0,8 mm 6,1 ± 1,8 mm
van Woerden et al.^4^	IC Controles	64 20	70 ± 10,7 66 ± 5,5	63 65	29,6 ± 5,7 27,2 ± 4,6	54,3 ± 8,5 59,7 ± 5,4	107,0 ± 27,7 (mL/m^2^) 76,9 ± 11,5 (mL/m^2^)

*IMC: Índice de massa corporal; CMD: Cardiomiopatia dilatada; TAE: Tecido adiposo epicárdico; FE: Fração de ejeção; IC: Insuficiência cardíaca; ICFEP: Insuficiência cardíaca com fração de ejeção preservada; ICFER: Insuficiência cardíaca com fração de ejeção reduzida; NR: Não relatado. Todos os estudos adotaram um nível de significância de p-valor <0,05.*

### Análise estatística

A associação entre TAE e IC foi medida por Diferença média padronizada (DMP) com IC 95%, devido ao uso de unidades de medição diferentes nos estudos. Em seguida, foram determinados os erros-padrão dos IC 95% correspondentes ou obtidos diretamente do estudo. O método do inverso da variância foi usado para ponderar os estudos quanto às estatísticas gerais combinadas. A significância estatística foi definida a um p-valor <0,05. A heterogeneidade entre os estudos foi avaliada utilizando-se as estatísticas do teste Q de Cochran e I^2^ e em seguida avaliada pelos valores I^2^. Valores de I^2^ abaixo de 30% foram definidos como heterogeneidade baixa; abaixo de 60% foram considerados como de heterogeneidade moderada; e acima de 60% foram considerados como de heterogeneidade alta.^11^ O modelo de efeitos aleatórios foi escolhido com base em diferenças na população dos estudos, que incluía vários fenótipos de IC, pacientes com várias comorbidades, e de inúmeros países. Foi realizada uma análise de sensibilidade excluindo estudos e verificando a consistência da estimativa de efeito geral. Não foi feita uma metarregressão devido ao pequeno número de estudos incluídos. Os resultados são relatados em gráficos de floresta com IC 95%. Todas as análises foram feitas com o software Review Manager 5.3.

## Resultados

### Seleção de estudos

**Figura 1:**
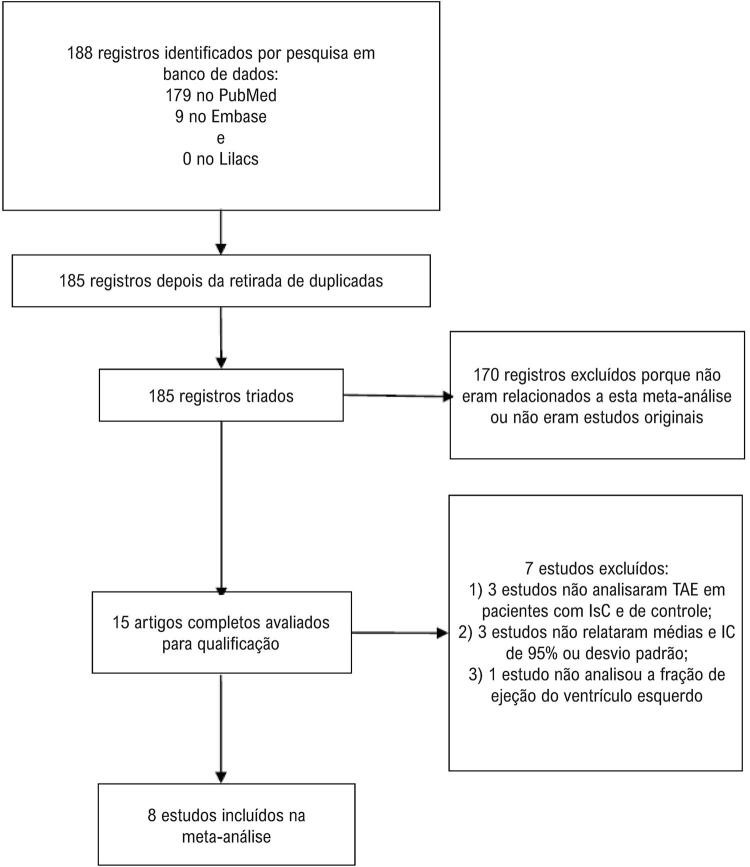
– Fluxograma da seleção dos estudos.

Inicialmente, um total de 188 estudos foram identificados nos bancos de dados, 179 no MEDLINE, 9 no Embase, e 0 no banco de dados Lilacs. Na análise de duplicatas, foram identificadas 3 duplicatas, que foram excluídas. Após uma leitura cuidadosa dos títulos e resumos, 170 dos 185 estudos foram excluídos por não estarem relacionados à presente revisão ou não serem estudos originais. Textos completos dos 15 estudos restantes foram analisados, e 8 deles foram incluídos na metanálise. Dos 7 estudos excluídos, 3 foram excluídos porque não analisavam o TAE em pacientes com IC e de controle, 3 foram excluídos porque não relatavam médias e IC 95% ou desvio padrão do TAE em pacientes com IC e no grupo de controle, e 1 foi excluído porque não analisava o FEVE. O fluxograma da seleção dos estudos está ilustrado na Figura 1.

### Características dos estudos incluídos

Foram incluídos oito estudos ^4 , 7 , 9 , 12 - 16^ nesta revisão, dos quais sete eram estudos observacionais prospectivos de centro único, enquanto um era um estudo observacional retrospectivo de centro único ^14^ (Tabelas 1 e 2).

No total, 1248 pacientes foram incluídos em nossa metanálise, dos quais 574 eram de controle, e 674 pacientes eram portadores de IC. Dos 674 pacientes com IC incluídos, 415 tinham IC com fração de ejeção reduzida (ICFER), e 259 tinham ICFEP ou ICFEfm. Quatro estudos ^7 , 9 , 12 , 13^ utilizaram a RMC para medir o TAE, dois estudos ^14 , 16^ utilizaram o ecocardiograma, um estudo ^15^ utilizou tomografia computadorizada (TC), e um estudo utilizou RMC e ecocardiograma para avaliar o TAE. ^4^ Além disso, estudos utilizaram pontos de corte de FEVE diferentes para definir os fenótipos de IC, conforme mostrado em detalhe nas Tabelas 1 e 2.

### Qualidade dos estudos incluídos

Todos os oito estudos incluídos nesta revisão foram classificados como bons pela NEWCASTLE - OTTAWA QUALITY ASSESSMENT SCALE CASE CONTROL STUDIES, tendo recebido sete ou oito estrelas em nove possíveis, conforme mostrado na Tabela 1.

### Associação de tecido adiposo epicárdico e insuficiência cardíaca

**Figura 2:**
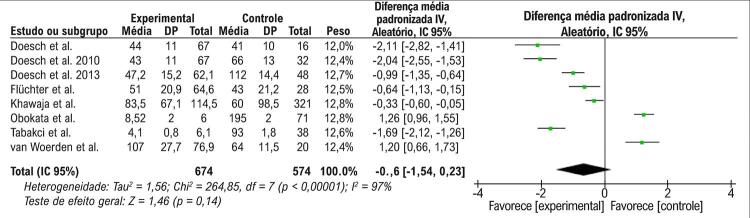
– Gráfico de floresta mostrando que a quantidade de TAE não está associada a todos os tipos de IC. Na coluna de Diferença média padronizada, os pontos vermelhos à esquerda da linha vertical representavam os estudos que encontraram uma quantidade reduzida de TAE na IC. Os pontos vermelhos à direita da linha vertical representavam os estudos que encontraram uma quantidade aumentada de TAE na IC. O losango preto representa a análise agrupada, e, como ele toca a linha vertical, o resultado não é estatisticamente significativo. Qui2: estatísticas de Qui-quadrado; IC: intervalo de confiança; gl : graus de liberdade; IC: insuficiência cardíaca; I2 : estatística de heterogeneidade de I quadrado; IV: inverso da variância; p : p-valor; DP: desvio padrão; EP: erro padrão; Std: padrão; Z: estatística Z.

**Figura 3:**
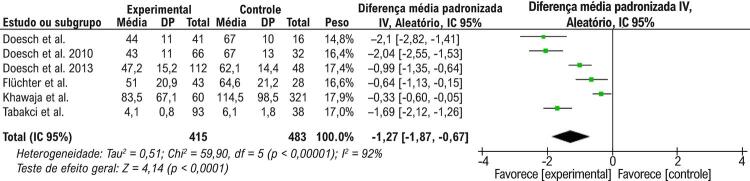
Gráfico de floresta mostrando que a quantidade de TAE é reduzida na ICFER. Na coluna de Diferença média padronizada, os pontos vermelhos à esquerda da linha vertical representavam a quantidade de TAE é reduzida na ICFER. O losango preto não toca a linha vertical demonstrando significância estatística. Qui2 : estatísticas de Qui-quadrado; IC: intervalo de confiança; gl : graus de liberdade; IC: insuficiência cardíaca; I2 : estatística de heterogeneidade de I quadrado; IV: inverso da variância; p : p-valor; DP: desvio padrão; EP: erro padrão; Std: padrão; Z: estatística Z.

**Figura 4:**
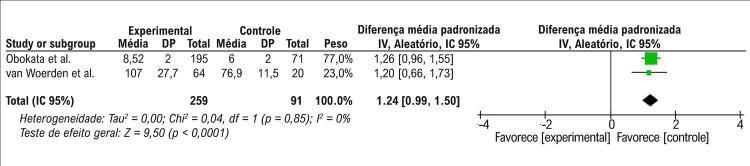
Gráfico de floresta mostrando que a quantidade de TAE está associada a ICFEP ou ICFEfm. Na coluna de Diferença média padronizada, os pontos vermelhos à direita da linha vertical representavam a quantidade de TAE é aumentada nos fenótipos de ICFEP e ICFEfm. O losango preto não toca a linha vertical demonstrando significância estatística. Qui2: estatísticas de Qui-quadrado; IC: intervalo de confiança; gl : graus de liberdade; I2 : estatística de heterogeneidade de I quadrado; IV: inverso da variância; p : p-valor; DP: desvio padrão; EP: erro padrão; Std: padrão; Z: estatística Z.

Nas análises de efeito aleatório, a quantidade de TAE não estava associada à IC quando todos os fenótipos de IC eram analisados (DMP = -0,66, IC 95%: -1,54 a 0,23, p =0,14), conforme apresentado na Figura 2. O teste de heterogeneidade demonstrou que havia diferenças significativas entre esses estudos (p<0,00001, I^2^=97%). Foi realizada uma análise de sensibilidade; entretanto, não foi possível retirar a heterogeneidade da metanálise. Realizando uma análise de subgrupo e avaliando a associação entre a quantidade de TAE com ICFER, nossa metanálise demonstrou que o TAE era significativamente mais baixo em pacientes com ICFER que nos do grupo de controle (DMP= -1,27, IC 95%: - 1,87 a -0,67, p <0,0001), como mostrado na Figura 3. O teste de heterogeneidade demonstrou que havia diferenças significativas entre esses estudos (p<0,00001, I^2^=92%). Embora tenha sido realizada uma análise de sensibilidade, não foi possível retirar a heterogeneidade da metanálise. Além disso, outra análise de subgrupo foi realizada para avaliar a associação entre a quantidade de TAE e ICFEP ou ICFEfm. Nesta metanálise, o volume de TAE era maior nos pacientes com ICFEP e ICFEfm que no grupo de controle (DMP 1,24, IC 95%: 0,99 a 1,50, p <0,0001), conforme apresentado na Figura 4. O teste de heterogeneidade demonstrou que havia diferenças significativas entre esses estudos (p = 0,85, I^2^=0%).

## Discussão

Até onde sabemos, esta é a primeira metanálise que investigou a associação entre TAE e IC. Em nossa análise, não houve relação significativa entre TAE e todos os tipos de IC. Entretanto, em uma análise de subgrupo incluindo apenas pacientes com ICFER, o volume de TAE era significativamente reduzido nos pacientes com ICFER em comparação ao grupo de controle. Além disso, após realizar uma análise incluindo pacientes com ICFEP ou ICFEfm, nossos resultados demonstraram que a quantidade de TAE era significativamente maior no grupo com ICFEP ou ICFEfm que nos controles.

### Anatomia e medição do tecido adiposo epicárdico

O TAE é um tecido adiposo visceral com correlação anatômica e fisiológica próxima ao miocárdio e as artérias coronárias. Ele se localiza atrás do pericárdio visceral, em contato direto com o músculo cardíaco, sem fáscia entre eles, partilhando a mesma vascularização coronária.^17 , 18^ Estudos anteriores também demonstraram que esse depósito de gordura está associado aos níveis de índice de massa corporal, circunferência de cintura, resistência à insulina, e outros traços de síndrome metabólica, mas também pode contribuir para a inflamação sistêmica além dos fatores de risco cardiovascular tradicionais.^19 , 20 , 21^ Portanto, devido à relação anatômica próxima entre TAE, o miocárdio e as artérias coronárias, o TAE pode ter um impacto na fisiopatologia de doenças cardiovasculares, tais como doença arterial coronariana, fibrilação atrial, e IC.^18 , 22^

As imagens por ressonância magnética (IRM), a TC, e o ecocardiograma podem avaliar adequadamente a quantidade de TAE, sendo que a IRM é o padrão-ouro.^23^ Embora as medições ecocardiográficas tenham uma boa correlação com a IRM, a variação espacial na janela ecocardiográfica é uma questão crítica que merece atenção na avaliação da espessura do TAE.^23^ Para minimizar esse problema, marcos anatômicos, tais como a posição do septo interventricular e o anel aórtico, devem sempre ser utilizados ao avaliar a espessura do TAE com o ecocardiograma.^23^

### Tecido adiposo epicárdico e insuficiência cardíaca com fração de ejeção reduzida

Em nossa metanálise, identificamos que, em pacientes com ICFER, o TAE era significativamente reduzido em comparação aos controles. Para pacientes com ICFER, o TAE pode ser reduzido devido a disfunção sistólica e altos níveis de peptídeo natriurético tipo B (BNP) nesse fenótipo. Na verdade, quando o miocárdio se torna disfuncional, ele desenvolve necessidades metabólicas anormais.^24 , 25^ Dessa forma, o papel do TAE como fonte de energia ou homeostase de citocinas diminuiria e, portanto o TAE é menos identificado.^24 , 25^ Além disso, em pacientes com ICFER com fração de ejeção reduzida grave, o volume de TAE reduzido pode indicar uma diminuição da capacidade de amortecimento de ácidos graxos livres (AGL) em excesso, além de uma capacidade mais baixa de se ajustar a demandas de energia especiais do coração disfuncional.^7^

Outro caminho possível para a redução de TAE é, na realidade, os peptídeos natriuréticos são capazes de estimular a lipólise nos adipócitos.^24 , 26 - 28^ Como os pacientes com ICFER geralmente têm níveis altos de BNP,^16^ mesmo quando comparados aos pacientes com ICFEP e ICFEfm, isso pode levar a quantidades mais baixas de TAE em pacientes com ICFER, conforme observado neste estudo.

Tecido adiposo epicárdico e insuficiência cardíaca com fração de ejeção na faixa média ou preservada

Nesta metanálise, pacientes com ICFEP e ICFEfm demonstraram uma quantidade mais alta de TAE, significativamente diferente em comparação aos pacientes de controle. Os resultados deste estudo estão em conformidade com uma metanálise anterior que demonstra uma associação entre TAE e disfunção diastólica,^29^ uma disfunção cardíaca que pode levar a ICFEP e também está presente nesses pacientes.

Diferentemente do ICFER, a ICFEP é uma síndrome altamente heterogênea e, até esta data, não existem tratamentos que possam reduzir efetivamente o risco de mortalidade em pacientes com essa doença.^30^ Um modelo fisiopatológico emergente de ICFEP demonstra que esse fenótipo de IC advém de uma inflamação sistêmica induzida por comorbidades que leva à disfunção endotelial, fibrose miocárdica, e rigidez de cardiomiócitos, que acabam resultando em ICFEP.^31^

Fisiologicamente, o TAE promove vários efeitos cardíacos protetores, devido a suas propriedades antiateroscleróticas e anti-inflamatórias, além de altos índices de liberação e captação de AGL.^32^ Entretanto, nesse modelo de inflamação induzida por comorbidades, obesidade, diabetes e outras comorbidades que são comuns em pacientes com ICFEP pode aumentar o TAE de maneira anormal, sobrecarregando as células adiposas.^7 , 33^ Consequentemente, essas células adiposas disfuncionais começam a liberar adipocinas pró-inflamatórias em circulação, que podem levar a um estado inflamatório sistêmico crônico.^5 , 34^ Esse estado está associado a várias alterações, tais como a rigidez arterial, disfunção endotelial das arteríolas, e fibrose.^4^

Além disso, como o TAE está localizado abaixo do pericárdio, ele poderia causar uma influência notável na função diastólica,^18 , 29^ devido aos efeitos restritivos mecânicos, reduzindo a distensibilidade do VE, que restringe seu enchimento e capacidade funcional.^35 - 40^

### Instruções adicionais

Embora essa metanálise demonstre que a quantidade de volume de TAE está associada à ICFEP e à ICFEfm, o estado inflamatório, e a secreção de citocinas pró-inflamatórias desse depósito de gordura na IC devem ser investigados. Além disso, estudos que investigam se alterações no volume de TAE estão associados a alterações na dinâmica do ventrículo esquerdo vão elucidar a relação entre TAE e função cardíaca. Além disso, são necessários estudos translacionais e ensaios clínicos que estudam terapias e intervenções com foco no TAE.

### Limitações

Esta metanálise tem várias limitações. Primeiro, como nossa metanálise só incluiu estudos observacionais, ela traz um viés inerente de desenho de estudo. Além disso, para analisar todas as evidências disponíveis, nossa metanálise incluiu estudos que usam métodos de imagem diferentes que avaliam o TAE, que poderiam ter contribuído para a alta heterogeneidade encontrada em nossos resultados. O fato de que os estudos incluídos analisaram pacientes de diferentes continentes e várias etiologias de IC poderia ter levado à heterogeneidade significativa observada nos resultados deste estudo. Finalmente, a análise de subgrupo de pacientes com ICFEP e ICFEfm incluiu um pequeno número de estudos, que destaca a necessidade de estudos posteriores que oferecem novos insights sobre essa associação.

## Conclusão

Em nossa análise, não houve relação significativa entre TAE e IC. Entretanto, em uma análise de subgrupo pré-especificado incluindo apenas pacientes com ICFER, o volume de TAE era significativamente reduzido nos pacientes com ICFER em comparação ao grupo de controle. Além disso, após realizar uma análise incluindo pacientes com ICFEP ou ICFEfm, o TAE era significativamente mais alto no grupo com ICFEP ou ICFEfm que nos controles. Portanto, o TAE poderia ser mais estudado em estudos translacionais para entender melhor a fisiopatologia da ICFEP e ICFEfm e, possivelmente, oferecer um novo alvo terapêutico para os tratamentos ICFEP e ICFEfm.
