# Supplementary material for: Tecido Adiposo Epicárdico nos Fenótipos de Insuficiência Cardíaca – Uma Metanálise
Source: Arq Bras Cardiol. 2022 Jan 11;118(3):625–33. [Article in Portuguese] doi: 10.36660/abc.20200755 (PMC8959032; doi:10.36660/abc.20200755)
Supplement: Translation

# Translation: Epicardial Adipose Tissue in Heart Failure Phenotypes – A Meta-Analysis

**Authors:** Eduardo Thadeu de Oliveira Correia, Letícia Mara dos Santos Barbetta, Orlando Santos da Costa, Pedro el Hadj de Miranda, Evandro Tinoco Mesquita

**Affiliations:** 1 Brazil Universidade Federal Fluminense, Niterói, RJ - Brazil

**Keywords:** Heart Failure/physiopathology, Pericardium/diagnostic, imaging, Adipose Tissue, Cytotoxins, Metanalisis

## Abstract

**Background:**

Epicardial adipose tissue (EAT) is increased in comorbidities common in heart failure (HF). In this sense, EAT could potentially mediate effects that lead to an impaired cardiac function.

**Objectives:**

This meta-analysis aims to investigate if the amount of EAT in all-types of HF and each HF phenotype is significantly different from control patients.

**Methods:**

This meta-analysis followed the Meta-analysis Of Observational Studies in Epidemiology guidelines. The search was performed in the MEDLINE, Embase, and Lilacs databases until November 2020. Two authors performed screening, data extraction, and quality assessment. A p-value <0.05 was defined as statistically significant.

**Results:**

Eight observational studies were included, comprehending 1,248 patients in total, from which 574 were controls, 415 had HF with reduced ejection fraction (HFrEF) and 259 had HF with mid-range or preserved ejection fraction (HFmrEF or HFpEF). The amount of EAT was not different between all types of HF and the control group (SMD = -0.66, 95% CI: -1.54 to 0.23, p =0.14). Analyzing each HF phenotype separately, patients with HFrEF had a reduced EAT when compared to the controls (SMD= -1.27, 95% CI: - 1.87 to -0.67, p <0.0001), while patients with HFmrEF or HFpEF showed an increased EAT when compared to controls (SMD= 1.24, 95% CI: 0.99 to 1.50, p <0.0001).

**Conclusion:**

The amount of EAT was not significantly different between all types of HF and the control group. In patients with HFrEF, the EAT volume was reduced, whereas in HFpEF and HFmrEF, the amount of EAT was significantly increased. PROSPERO registration number: CRD42019134441.

## Introduction

Epicardial adipose tissue (EAT) is a visceral fat depot localized around the myocardium.^1 , 2^ EAT secretes several proinflammatory chemokines and cytokines, collectively called adipokines.^3^ In addition, due to the close relation of EAT and the myocardium, epicardial fat may promote mechanical and local inflammatory effects on the cardiac muscle and coronary vessels.^4^

It is also known that EAT is increased in systemic diseases, which can promote a systemic proinflammatory state, such as obesity and diabetes, common in heart failure (HF) patients, mainly in HF with preserved ejection fraction (HFpEF).^5 - 7^ In this sense, EAT may mediate deleterious effects on the myocardium, which can lead to an impaired cardiac function.^8^

Previous studies have shown that EAT is lower in HF patients than in healthy patients.^9^ However, a recent study published by van Worden et al., which submitted 64 patients with HF with mid-range ejection fraction (HFmrEF) or HFpEF to cardiac magnetic resonance (CMR) showed that HF patients had a higher EAT volume than the control group.^4^ Despite the relevance of this association, there are no systematic reviews or meta-analyses that weigh the available evidence and bring conclusions and discussions about this topic. Therefore, the present study aims to perform a meta-analysis to investigate the association between EAT and each HF phenotype.

## Methods

A meta-analysis was performed using the criteria established by the Meta-analysis of Observational studies in the Epidemiology Group (MOOSE) recommendations.^10^ The protocol of this meta-analysis was registered in PROSPERO, logged under the following registration number: CRD42019134441.

### Search strategies

Two investigators (ETOC, LMSB) searched the MEDLINE, the Lilacs, and the Embase database for studies that investigated EAT in patients with HF, until November 2020. The search strategy was made by a combination of English terms and Medical Subject Heading (MeSH) descriptors, consisting of four keywords [(epicardial adipose OR epicardial fat) AND (heart failure OR cardiac insufficiency)]. A manual search of references was also used to identify possible studies for inclusion. Each title and abstract were independently analyzed by the two investigators, who selected the articles that would be relevant to the review. After, full texts of the remaining articles were reviewed to select which would be included in the quantitative analysis. In case of disagreement, the decision was made by discussion and consensus of the authors.

### Inclusion criteria

Eligible studies were required to meet the following criteria: 1) Population: the study included human subjects with HF; 2) Intervention: the study measured EAT and left ventricular ejection fraction (LVEF) with computerized tomography, echocardiography, or CMR; 3) Comparison group: the study included patients without HF; 4) Outcomes: the study reported means and 95% confidence intervals (CI) or standard deviation of EAT in patients with HF and the control group; 5) Study Design: this was an observational study.

### Data extraction

Data extraction was performed by two investigators, using a standard form (PMH, OSC), and cross-checked by a third author (ETOC). Extracted data included: 1) First author’s last name, publication year; 2) Characteristics of the included studies: number of patients, country of the study, study arms, etiology of HF, New York Heart Association class of the included patients, the ejection fraction of the included patients, age, body mass index, method of EAT measurement, and main findings 3) Outcome results: means, 95% CI, and standard deviation of EAT in patients with HF, and the control group.

### Quality assessment

The risk of bias in the studies was evaluated by the NEWCASTLE - OTTAWA QUALITY ASSESSMENT SCALE CASE CONTROL STUDIES, which analyzes the selection of participants in each study, the comparability of cases and controls, and the exposure. Quality assessment was performed by two investigators, using a standard questionnaire (PMH, OSC), in case of divergence, a conclusion was reached by consensus. The quality of studies was considered good if they scored between 7-8 points, satisfactory if they scored between 5-6 points, and unsatisfactory if they scored from 0 to 4 points. Quality assessment of the included studies is reported in Tables 1 and 2.

**Table 1:** – Characteristics of the included studiess

Study	Country	Definition of HF# and/or HF# subtypes	Control group definition	Measurement method	Quality
Doesch et al.^12^	Germany	Presence of LVEF ≤35% on echocardiography and signs and symptoms of HF. These patients were classified in HF due ICM or DCM.	Healthy patients	CMR	Good
Doesch et al.^9^	Germany	Patients with LVEF <50% on echocardiography in the absence of significant CAD (coronary artery stenosis ≥ 50% or history of coronary revascularization or previous MI).	Healthy patients	CMR	Good
Doesch et al.^7^	Germany	History of symptomatic HF and LVEF ≤ 35% on echocardiography. The ischemic etiology was defined as the presence of any epicardial coronary vessels with ≥75% stenosis or history of MI or coronary revascularization. DCM was based on end-diastolic diameter >56mm and a normal coronary angiography performed within the previous 6 months.	Healthy patients	CMR	Good
Flüchter et al.^13^	Germany	Patients with previous HF subclassified in ischemic and dilated etiologies.	Healthy patients	CMR	Good
Obokata et al.^14^	The United States	HFpEF was defined by clinical symptoms of HF, LVEF >=50%, directly measured elevation in LV filling pressures (at rest>15mmHg and/or with exercise ≥25mmHg). Non-obese HFpEF was defined by BMI <30kg/m^2^. Obese HFPEF was defined by the presence of class II or greater obesity (BMI ≥35kg/m^2)^.	Non-obese patients free of HF	Echocardiography	Good
Khawaja et al.^15^	The United States	Patients with LVEF ≤55%. This group was subdivided into patients with moderate LV dysfunction (LVEF 35% to 55%) and a group with severe LV dysfunction (LVEF ≤55%).	Patients without history of HF or LV dysfunction on echocardiogram	CT	Good
Tabakci et al.^16^	Turkey	NICMP was defined as LVEF ≤45 with normal coronary epicardial arteries seen in angiography.		CMR	Good
van Woerden et al.^4^	The Netherlands	Patients with LVEF >= 40% on echocardiography, NT-proBNP >125ng/L and echocardiographic evidences of LV hypertrophy, LV diastolic dysfunction, or left atrial dilatation according to ESC criteria. HFmrEF were defined by LVEF 40–50 and HFpEF patients were defined by LVEF >50%.	Healthy patients	CMR	Good

*BMI: body mass index; CAD: coronary artery disease; CMR: cardiac magnetic resonance; CT: computerized tomography; DCM: dilated cardiomyopathy; HF: heart failure; HFmrEF: heart failure with mid-range ejection fraction; HFpEF: heart failure with preserved ejection fraction; HFrEF: heart failure with reduced ejection fraction; ICM: ischemic cardiomyopathy; LV: left ventricle; LVEF: left ventricular ejection fraction; MI: myocardial infarction; NICMP: nonischemic dilated cardiomyopathy; NT-proBNP: N-terminal pro-B-type natriuretic peptide; NYHA: New York Heart Association.*

**Table 2:** – Characteristics of the included studies (continued)

Study	Arms	N	Age, year	Male Sex (%)	BMI	EF	EAT| volume, weight or thickness
Doesch et al.^12^	HF (EF ≤ 35%) Controls	41 16	63 ± 12 61 ± 11	88 75	27 ± 4 28 ± 5	27 ± 9 57 ± 6	44 ± 11 (g) 67 ± 10 (g)
Doesch et al.^9^	DCM (EF 35-50%) DCM (EF ≤35%) All DCM Controls	28 84 112 48	57,2 ± 13,4 60,1 ± 14,0 59,4 ± 13,9 60,9 ± 9,8	79 77 78 77	26,6 ± 4,6 27,3 ± 4,8 27,2 ± 4,7 27,3 ± 6,0	43,6 ± 6,9 23,0 ± 6,7 58,7 ± 5,2 58,7 ± 5,2	50.0 ± 21.9 (ml) / 25.0 ± 10.4 (ml/m^2^) / 47.0 ± 20.6 (g) / 23.5 ± 9.8(g/m^2^) 50.2 ± 13.9 (ml) / 25.7 ± 7.0 (ml/m^2^) / 47.2 ± 13.1 (g) / 24.2 ± 6.6 (g/m^2^) 50.2 ± 16.2 (ml) / 25.5 ± 8.0 (ml/m^2^) / 47.2 ± 15.2 (g) / 24.0 ± 7.5 (g/m^2^) 66.0 ± 15.3 (ml) / 33.5 ± 6.4 (ml/m^2^) / 62.1 ± 14.4 (g) / 31.7 ± 5.6 (g/m^2^)
Doesch et al.^7^	HF| Controls	66 32	63 ± 12 57 ± 11	82 78	27 ± 4 28 ± 4	27 ± 9 58 ± 5	46 ± 11 (ml) / 43 ± 11 (g) / 24 ± 5 (ml/m^2^) 71 ± 13 (ml) /67 ± 13 (g) / 36 ± 5 (ml/m^2^)
Flüchter et al.^13^	HF Controls	43 28	61,9±12,4 56,6 ± 10,9	81,3 78,6	27,0 ± 4,4 27,5 ± 4,2	25,9 ± 6,8 58,8 ± 4,2	51.0 ± 20.9 g / 3.5 ± 1.5(mm)- (Long-axis) / 2.9 ± 1.3(mm)-(Short-axis) /3.2 ± 1.2(mm)- (Longaxis/Short-axis) 64.6 ± 21.2 g / 3.8 ± 1.5(mm)- (Long-axis) / 4.3 ± 1.3(mm)-(Short-axis) /4.1 ± 1.1(mm)- (Longaxis/Short-axis)
Obokata et al.^14^	Controls Non-obese HFpEF (EF>50%) Obese HFpEF (EF>50%)	71 96 99	62 ± 10 70 ± 10 65 ± 11	42 36 36	25,4 ± 2,8 26,0 ± 2,7 40,8 ± 5,6	63 ± 4 63 ± 6 63 ± 6	6 ± 2 mm / 632 (517 - 768) ml 7±2 mm / 797 (643 - 979) ml 10±2 mm / 945 (831 - 1105) ml
Khawaja et al.^15^	HF (EF<55%) HF (EF 35-55%) HF (EF≤35%) Controls	60 43 17 321	54,2 ± 12,2 53,4 ± 12,2 59,8 ± 14,4 55,8 ± 10,4	19 35 53 70	30,7 ± 11,5 29,5 ± 4,7 31,3 ± 13,6 29,6 ± 6,7	NR NR NR NR	83.5 ± 67.1 (cm^3^) 96.1 ± 73.9 (cm^3^) 52.2 ± 29.7 (cm^3^) 114.5 ± 98.5 (cm^3^)
Tabakci et al.^16^	DCM Controls	93 38	49,9 ± 13,9 51,1 ± 10,0	69 61	27,7 ± 3,3 28,3 ± 3,4	32,0 ± 8,5 62,9 ± 4,9	4.1 ± 0.8 mm 6.1 ± 1.8 mm
van Woerden et al.^4^	HF Controls	64 20	70 ± 10,7 66 ± 5,5	63 65	29,6 ± 5,7 27,2 ± 4,6	54,3 ± 8,5 59,7 ± 5,4	107.0 ± 27.7 (mL/m^2^) 76.9 ± 11.5 (mL/m^2^)

*BMI: body mass index; DCM: dilated cardiomyopathy; EAT: epicardial adipose tissue; EF: ejection fraction; HF: heart failure; HFpEF: heart failure with preserved ejection fraction; HFrEF: heart failure with reduced ejection fraction; NR: not reported. All studies adopted a significance level of p-value < 0.05.*

### Statistical analysis

The association between EAT and HF was measured by Standard Mean Difference (SMD) with 95% CI, due to different units of measurement used across the studies. Subsequently, standard errors were determined from the corresponding 95% CIs or directly obtained from the study. The inverse variance method was used to weigh studies for the combined overall statistics. Statistical significance was defined at p-value < 0.05. Heterogeneity between studies was assessed using the Cochrane Q and I^2^statistics and then evaluated by I^2^values. I^2^values less than 30% were defined as low heterogeneity; less than 60% were considered moderate heterogeneity; and more than 60% were determined as high heterogeneity.^11^ The random-effects model was chosen based on differences in the population of the studies, which included different phenotypes of HF, as well as patients with several comorbidities and from numerous countries. A sensitivity analysis was performed by leaving out studies and checking the consistency of the overall effect estimate. A meta-regression was not performed because of the small number of studies included. The results are reported in forest plots with 95% CI. All analyses were done using Review Manager 5.3 software.

## Results

### Study selection

Initially, a total of 188 studies were identified in the databases, 179 in MEDLINE and 9 in Embase, and 0 in the Lilacs database. In the duplicate analysis, 3 duplicates were identified, which were excluded. After a careful reading of titles and abstracts, 170 of 185 studies were excluded because they were not related to the present review or were not original studies. Full texts of the remaining 15 studies were analyzed, and 8 of them were included in the meta-analysis. Of the 7 studies excluded, 3 were excluded because they did not analyze EAT in HF and control patients, 3 were excluded because did not report means and 95% CI or standard deviation of EAT in patients with HF and the control group, and 1 was excluded because it did not analyze LVEF. The flow diagram of the study selection is depicted in  Figure 1  .

**Figure 1:** – Flow diagram of the study selection.

### Characteristics of the included studies

Eight studies ^4 , 7 , 9 , 12 - 16^ were included in this review, seven of which were prospective single-center observational studies and one single-center retrospective observational study ^14^ (Tables 1 and 2).

Overall, 1,248 patients were included in our meta-analysis, of which 574 were controls and 674 patients with HF. Of the 674 HF patients included, 415 had HF with reduced ejection fraction (HFrEF) and 259 had HFpEF or HFmrEF. Four studies ^7 , 9 , 12 , 13^ used CMR to measure the EAT, two studies ^14 , 16^ used echocardiography, one study ^15^ used computerized tomography (CT), and one study used CMR and echocardiography to evaluate de EAT. ^4^ Studies have also used different LVEF cut-off points to define HF phenotypes, as shown in detail in Tables 1 and 2.

### Quality of the included studies

All eight studies included in this review were graded as good by the NEWCASTLE - OTTAWA QUALITY ASSESSMENT SCALE CASE CONTROL STUDIES, having received seven to eight stars out of nine, as shown in  Table 1  .

### Association of Epicardial Adipose Tissue and Heart Failure

In random-effects analyses, the amount of EAT was not associated with HF when all the phenotypes of HF were analyzed (SMD= -0.66, 95% CI: -1.54 to 0.23, p =0.14), as shown in  Figure 2  . The heterogeneity test showed that there were significant differences between studies (p<0.00001, I^2^=97%). A sensitivity analysis was performed; however, it was not possible to remove the heterogeneity from the meta-analysis.

**Figure 2:** – Forest plot showing that the amount of EAT is not associated with all-types of HF. In the Standard Mean Difference column, the red dots to the left of the vertical line represented the studies that found a reduced amount of EAT in HF. The red dots to the right of the vertical line represented the studies that found an increased amount of EAT in HF. The black diamond represents the pooled analysis, and because it touches the vertical line, the result is not statistically significant. Chi2: chi-square statistic; CI: confidence interval; df: degrees of freedom; HF: heart failure; I2: I-square heterogeneity statistic; IV: inverse variance; p : p value; SD: standard deviation; SE: standard error; Std: standard; Z: Z statistic.

Performing a subgroup analysis evaluating the association between the amount of EAT with HFrEF, our meta-analysis showed that EAT was significantly lower in HFrEF patients than in the control group (SMD= -1.27, 95% CI: - 1.87 to -0.67, p <0.0001), as shown in  Figure 3  . The heterogeneity test showed that there were significant differences between studies (p<0.00001, I^2^=92%). Although a sensitivity analysis was performed, it was not possible to remove the heterogeneity from the meta-analysis.

**Figure 3:** Forest plot showing that the amount of EAT is reduced in HFrEF. In the Standard Mean Difference column, the red dots to the left of the vertical line indicated that the amount of EAT is reduced in HFrEF. The black diamond does not touch the vertical line, showing statistical significance. Chi2 : chi-square statistic; CI: confidence interval; df : degrees of freedom; HF: heart failure; I2: I-square heterogeneity statistic; IV: inverse variance; p: p value; SD: standard deviation; SE: standard error; Std: standard; Z: Z statistic.

In addition, another subgroup analysis was performed to evaluate the association between the amount of EAT with HFpEF or HFmrEF. In this meta-analysis, the EAT volume was greater in HFpEF and HFmrEF than in the control group (SMD= 1.24, 95% CI: 0.99 to 1.50, p <0.0001), as shown in  Figure 4  . The heterogeneity test showed that there were no significant differences between studies (p = 0.85, I^2^=0%).

**Figure 4:** Forest plot showing that the amount of EAT is associated with HFpEF or HFmrEF. In the Standard Mean Difference column, the red dots on the right of the vertical line indicated that the amount of EAT is increased with HFpEF and HFmrEF phenotypes. The black diamond does not touch de vertical line showing statistical significance. Chi2 : chi-square statistic; CI: confidence interval; df : degrees of freedom; I2: I-square heterogeneity statistic; IV: inverse variance; p : p value; SD: standard deviation; SE: standard error; Std: standard; Z : Z statistic.

## Discussion

To the best of our knowledge, this is the first meta-analysis that investigated the association between EAT and HF. In our analysis, there was no significant relation between EAT and all-types of HF. However, in a subgroup analysis including only patients with HFrEF, the EAT volume was significantly reduced in HFrEF when compared to the control group. Furthermore, after performing an analysis including patients with HFpEF or HFmrEF, our results showed that the amount of EAT was significantly greater in the HFpEF or HFmrEF group than in the controls.

### Anatomy and Measurement of the Epicardial Adipose Tissue

EAT is a visceral adipose tissue with close anatomical and physiological correlation with the myocardium and coronary arteries. It is located behind the visceral pericardium, in direct contact with the cardiac muscle, with no fascia between them, sharing the same coronary vascularization.^17 , 18^ Previous studies have also shown that this fat depot is associated with body mass index levels, waist circumference, insulin resistance, and other metabolic syndrome traits, but may also contribute to systemic inflammation beyond traditional cardiovascular risk factors.^19 , 20 , 21^ Thus, due to the close anatomical relation among EAT, the myocardium, and coronary arteries, EAT may have an impact on the pathophysiology of cardiovascular diseases, such as coronary artery disease, atrial fibrillation, and HF.^18 , 22^

Magnetic Resonance Imaging (MRI), CT, and echocardiography can adequately assess the amount of EAT, MRI being the gold-standard.^23^ Although echocardiographic measurements have a good correlation with MRI, spatial variation in the echocardiographic window is a critical issue that needs attention when evaluating EAT thickness.^23^ To minimize this problem, anatomical landmarks, such as the position of the interventricular septum and the aortic annulus, should always be used when assessing EAT thickness with the echocardiogram.^23^

### Epicardial Adipose Tissue and Heart Failure with Reduced Ejection Fraction

In our meta-analysis, we found that in patients with HFrEF, EAT was significantly reduced when compared to the controls. For patients with HFrEF, EAT may be reduced due to systolic dysfunction and high levels of b-type natriuretic peptide (BNP) in this phenotype. In fact, when myocardium becomes dysfunctional, it develops abnormal metabolic needs.^24 , 25^ In this sense, the role of EAT as a source of energy or cytokine homeostasis would decrease, and the EAT would be less found.^24 , 25^ Moreover, in patients with HFrEF with a severely reduced ejection fraction, the reduced EAT volume may indicate a decreased buffering capacity for excess fatty free acids (FFA), as well as a lower capacity to adjust to special energy demands of the dysfunctional heart.^7^

Another possible pathway for EAT reduction is the fact that natriuretic peptides are capable of stimulating lipolysis in adipocytes.^24 , 26 - 28^ Since patients with HFrEF often have high levels of BNP,^16^ even when compared to HFpEF and HFmrEF, this might lead to lower amounts of EAT in HFrEF, as observed in this study.

### Epicardial Adipose Tissue and Heart Failure with Preserved or Mid-Range Ejection Fraction

In this meta-analysis patients with HFpEF and HFmrEF demonstrated a higher amount of EAT, which was significantly different when compared to the control patients. Our results are in agreement with a previous meta-analysis, which showed an association between EAT and diastolic dysfunction,^29^ a functional cardiac dysfunction that can lead to HFpEF and is also present in these patients. Differently from HFrEF, HFpEF is a highly heterogeneous syndrome and, to date, there are no therapies that can effectively reduce the risk of mortality in patients with this condition.^30^ An emerging pathophysiological model of HFpEF shows that this HF phenotype comes from a comorbidity-induced systemic inflammation that leads to endothelial dysfunction, myocardial fibrosis, and cardiomyocyte stiffening, which ultimately results in HFpEF.^31^

Physiologically, EAT promotes several protective cardiac effects, due to its anti-atherosclerotic and anti-inflammatory properties, as well as a high FFA release and uptake rates.^32^ However, in this comorbidity-induced inflammation model, obesity, diabetes, and other comorbidities that are common in patients with HFpEF may abnormally increase EAT, overloading fat cells.^7 , 33^ Consequently, these dysfunctional fat cells start to release pro-inflammatory adipokines into circulation, which can lead to a chronic systemic inflammatory state.^5 , 34^ This state is associated with several alterations, such as arterial stiffening, endothelial dysfunction of arterioles, and fibrosis.^4^

Moreover, since EAT is located under the pericardium, it could cause a remarkable influence on the diastolic function,^18 , 29^ due to mechanical restraint effects, reducing LV distensibility, which restricts LV filling and functional capacity.^35 - 40^

### Further Directions

Although this meta-analysis shows that the amount of EAT volume is associated with HFpEF and HFmrEF, the inflammatory state and the secretion of pro-inflammatory cytokines of this fat depot in HF must be investigated. Furthermore, studies that investigate if changes in EAT volume are associated with changes in left ventricular dynamics will elucidate the relation between EAT and cardiac function. Moreover, translational studies and clinical trials that study therapies and interventions targeting EAT are warranted.

### Limitations

This meta-analysis has several limitations. First, since our meta-analysis only included observational studies, it carries an inherent bias of this study design. Moreover, to analyze all evidence available, our meta-analysis included studies using different imaging methods that assessed EAT, which could have contributed to the high heterogeneity found in our results. The fact that included studies analyzed patients from different continents and several HF etiologies might also have led to the significant heterogeneity observed in our results. Finally, the subgroup analysis of patients with HFpEF and HFmrEF included a small number of studies, which highlights the need for further studies that provide new insights into this association.

## Conclusion

In our analysis, there was no significant relation between EAT and HF. However, in a prespecified subgroup analysis including only patients with HFrEF, the EAT volume was reduced in HFrEF when compared to the control group. In addition, after performing an analysis including patients with HFpEF or HFmrEF, EAT was significantly higher in HFpEF or HFmrEF than in the controls. Therefore, EAT could be further studied in translational studies to better understand the pathophysiology of HFpEF and HFmrEF and, possibly, provide a novel therapeutic target for HFpEF and HFmrEF therapies.
